# Physicochemical and functional properties of dried okra (*Abelmoschus esculentus L.*) seed flour

**DOI:** 10.1002/fsn3.1725

**Published:** 2020-06-30

**Authors:** Jemima Ofori, Charles Tortoe, Jacob K. Agbenorhevi

**Affiliations:** ^1^ Food Research Technology Division Council for Scientific and Industrial Research ‐ Food Research Institute Accra Ghana; ^2^ Department of Food Science and Technology Kwame Nkrumah University of Science and Technology Kumasi Ghana

**Keywords:** *Abelmoschus esculentus*, flour, functional properties, pasting properties, proximate composition

## Abstract

The physicochemical and functional properties of dried okra seed flour of two genotypes *Agbagoma* and *Balabi* were evaluated. The samples *Agbagoma* and *Balabi* had 8.90%–9.00% moisture, 16.80%–17.40% protein, 47.80%–48.00% fat, 7.70%–7.80% ash, and 18.20%–18.40% carbohydrate. The mean values of functional properties revealed significant differences (*p* < .05) between okra seed flour samples studied. The samples’ bulk density range was 0.80–0.83 g/ml. Water absorption capacity and oil absorption capacity ranged from 511.65% to 504.32% and 88.38 to 160.67%, respectively. The solubility was 14.10% for *Agbagoma* and 10.97% for *Balabi,* whereas swelling power was 16.37% and 14.68% for *Agbagoma* and *Balabi,* respectively. All pasting properties except peak time and pasting temperature of *Agbagoma* seed flour were higher than those of *Balabi* seed flour. The study revealed that dried okra seed flour is rich in nutrients, which could be used for baking and fortification of foods.

## Graphical abstract

The physicochemical and functional properties of dried okra seed flour of two genotypes Agbagoma and Balabi were evaluated. The mean values of physicochemical and functional properties revealed significant differences (*p* < .05) between the okra seed flour samples. 0kra seed flour from Balabi could be used as composite for baby foods due to its low viscosity whereas Agbagoma flour could be used as composite for high viscous food.

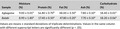

## 1 INTRODUCTION

Okra *(Abelmoschus esculentus L*.) is one of the most widely known and utilized species of the family *Malvaceae*. Economically, okra is an important vegetable crop grown in tropical and sub‐tropical regions of the world. Mostly, it is grown for its green leaves and pods as green vegetable according to Naveed, Khan, and Khan, (2009). In studies reported by Gopalan, Ramasastri, and Balaubramanian, (2007), one hundred grams (100 g) of okra contain moisture (89.6 g), minerals (0.7 g), protein (1.9 g/100 g), carbohydrates (6.4 g), fat (0.2 g), calcium (66 mg), fiber (1.2 g), calories (35 mg), potassium (103 mg), phosphorus (56 mg), magnesium (53.0 mg), and sodium (6.9 mg). Additionally abundant in okra are several vitamins such as vitamin C (21.1 mg), vitamin A (375 IU), vitamin E (0.36 mg), vitamin K (53 ug), thiamine (0.200 g) of fresh, raw pods value per 100 g (USDA, 2018) and minerals such as calcium (111.11–311.95 mg/100 g), iron (18.30–36.68 mg/100 g), potassium (122.59–318.20 mg/100 g), zinc (3.83–6.31 mg/100 g), phosphorus (25.62–59.72 mg/100 g), and sodium (3.33–8.31 mg/100 g) on dry weight bases (Gemede, Haki, Beyene, Woldegiorgis, & Rakshit, 2016).

In traditional alternative medicine treatment, it has been used in the treatment of diabetes based on the presence of large amount of soluble dietary fibers which retard glucose absorption from the gastrointestinal tract (Khatun, Rahman, Biswas, & Islam, 2011). Okra seed is rich in high‐quality protein especially its essential amino acids compared with other plant protein sources (Gemede, 2016). According to Arapitsas (2008), Manach, Williamson, Morand, Scalbert, and Remesy (2005), Ngoc, Ngo, Van, and Phung (2008), okra seed contains oligomeric catechins (2.5 mg/g of seeds) and flavonol derivatives (3.4 mg/g of seeds) as well as hydroxycinnamic and quercetin derivatives (0.2 and 0.3 mg/g of skins) in the mesocarp. Further, okra seeds contain about 20 to 40% oil (Arapitsas, 2008; Manach et al., 2005; Ngoc et al., 2008) and also rich in antioxidants, which are essential in maintaining health (Graham, Agbenorhevi, & Kpodo, 2017). The chemical composition of okra seed flour revealed a predominance of moisture (6.96%), total carbohydrates (30.81%), protein (22.14%), oil (14.01%), and crude fiber (27.30%) (Moyin‐Jesu, 2007).

Okra pods can be consumed in many ways as fresh (raw), dried, cooked, frozen, fried, and pickled. Utilization of okra seeds is numerous. In Turkey, mature okra seeds are roasted, ground, and used as a coffee substitute (Calisir & Yildiz, 2005). In Africa, often okra seeds are dried and used to prepare vegetable curds (Moekchantuk & Kumar, 2004). Adelakun and Oyelade (2011) opined that okra seed flour has huge potential of being used to enrich foods to improve daily nutritional needs. Okra has industrial applications and is used in confectionary products (Adetuyi, Badejo, Ikujenlola, & Omosuli, 2009). In Egypt, okra seed flour has been used to supplement corn flour to produce better quality dough (Kumar, Patil, Patil, & Paschapur, 2009).

In Ghana, the major okra genotypes identified in Ghana with high seed contents include *Agbagoma* and *Balabi*. However, literature is limited on the physicochemical and functional properties of the seed flour to explore alternative uses in food systems. In addition, pectin is commercially extracted from the okra pods (Kpodo et al., 2017) leaving the seeds wasted, which can be utilized in the production of seed flour. The objective of this work was to determine the physicochemical and functional properties of the dried seed flour of *Agbagoma* and *Balabi* genotypes cultivated in Ghana.

## 2 MATERIALS AND METHODS

### 2.1 Materials and reagents

All chemicals used were analytical grade reagents. Two okra genotypes *Agbagoma* and *Balabi* were cultivated at Akrofu in the Volta Region of Ghana, and all agricultural practices including thinning, weed control, and watering were carried out under controlled environmental conditions. The okra pods were harvested after 3 months of cultivation.

### 2.2 Preparation of okra seed flour

Harvested *Agbagoma* and *Balabi* okra genotypes were sorted, cleaned, and washed with distilled water and cut opened using a stainless knife to remove seeds. Seeds were then sun‐dried for 6 hr. The sun‐dried seeds were ground into powder of particle size less than 450 µm using a grinding mill (Christy and Norris Ltd., Surrey, UK) and packaged in zip‐lock bags and stored at −20°C in a freezer (Protech PRCF‐500, China) for further analysis.

### 2.3 Proximate analysis

Proximate composition of moisture, crude protein, ash, and crude fat of the dried okra seed flour samples was determined according to AOAC (2005). Protein was calculated from total nitrogen using the conversion factor 6.25. The percentage of total carbohydrate content of okra seed flour sample was calculated by subtracting the percentage of moisture, ash, protein, and fats obtained from 100.

### 2.4 Determination of pH, color, and bulk density

The pH was determined by the method as described by AOAC (2005). Two grams (2.0 g) of okra seed flour was poured into three beakers containing 20 ml of distilled water and allowed to stand for a while and an electric digital pH meter (BECKMAN Φ340 pH/Temp. Meter) was used to determine the pH of the samples. The pH meter was dipped into the sample and the reading was taken after about 4 min when it was stable.

The color of the okra seed flour was measured using the CR‐400 Chroma Meter which is a handheld, portable measurement instrument designed to evaluate the color of objects.

The color meter coordinates system L* a* b* values were recorded, and the Light index was calculated as (100/0). The white tile used for calibrating the Hunter L* a* b* color scale had L* = + 97.51, a* = + 0.29 and b* = 1.90 as standards.

Bulk density was determined by modifications of the gravimetric method by Asoegwu, Ohanyere, Kanu, and Lwueke (2006). Fifty grams (50 g) of okra seed flour sample was weighed into 100 ml measuring cylinder. The bottom of the cylinder was tapped repeatedly on a firm pad on a laboratory bench until a constant volume was observed. The packed volume was recorded. The bulk density (BD) was calculated by using a ratio of sample weight to constant volume obtained as:

(1) $$\text{Bulk Density}=\frac{\text{Weight of sample}}{\text{constant volume}}$$

### 2.5 Determination of water and oil absorption capacities

Water and oil absorption capacity was determined by the method of Beuchat (1977). One gram (1 g) of okra seed flour sample was mixed with 10 ml distilled water for 30 s. The samples were then allowed to stand at room temperature (25 ± 2°C) for 30 min after which they were centrifuged at 3,000 rpm for 30 min. The volume of the supernatant was noted in a 10 ml graduated cylinder. Water absorption capacity (mg.mL^‐1^) was calculated as the difference between the initial volume of water added to the sample and the volume of the supernatant. The same procedure was carried out to determine the oil absorption capacity as below;

(2) $$\text{Water Absorption Capacity}/\text{Oil Absorption Capacity}=\frac{\text{Bound water}/\text{oil}}{\text{weight of sample}}\times 100$$

### 2.6 Determination of swelling power and water solubility index

Swelling power and water solubility indices were carried out based on a modification of the method of Leach, McCoven, and Scoch (1959). One gram (1 g) of sample was transferred into a weighed graduated 15 ml centrifuge tube. Ten milliliters (10 ml) of distilled water was added. The suspension was stirred sufficiently and uniformly with a stirrer avoiding excessive speed. The sample was heated at 85°Ϲ in a thermostatically regulated temperature bath for 30 min with constant stirring. The tubes were removed wiped dry on the outside and cooled to room temperature. It was then centrifuged for 15 min at 2,200 rpm in a centrifuge. The solubility was determined by evaporating the supernatant in a hot air oven at 105°Ϲ (Gallenkamp, England) and weighed. The swelling volume was obtained by directly reading the volume of the swollen sediment in the tube. The sediment paste was weighed. The swelling power and water solubility index were calculated using the relations:

(3) $$\begin{array}{cc} \text{Swelling Power} & =\frac{\text{wt of precipitated paste}}{\text{wt of sample}}\\ & \;-\text{wt of residue in supernatant} \end{array}$$

(4) $$\text{Solubility Index}\left(\%\right)=\frac{\text{Weight of soluble fraction}}{\text{Weight of sample}}\times 100$$

### 2.7 Determination of pasting properties

Rapid Viscos Analyzer (RVA Model 4,500, Perten Instruments, Australia) was used in the analysis of the pasting properties of the dried okra seed flour samples. The calculated moisture content was inputted into the RVA which gives the mass of flour and water to be measured. The masses displayed by the instrument were measured into a dried empty canister. The dispersion was mixed thoroughly and then placed into the RVA. The slurry was heated from 50°Ϲ to 95°Ϲ.

### 2.8 Statistical analysis

Two samples independent test was used for mean comparison to identify significant differences between the samples. Statistical significance was accepted at 95% confidence limit (*p* < .05).

## 3 RESULTS AND DISCUSSION

### 3.1 Proximate composition of okra seed flour

Moisture content of the okra seed flour samples ranged from 8.90% to 9.00% (Table 1). The *Balabi* had the lower average moisture content than *Agbagoma* genotype. The moisture content of flour studied (8.9%–9.0%) falls within the acceptable range of 0%–13% (James, 1995). This moisture content range has been reported to be unfavorable for spoilage organisms to thrive (James, 1995), therefore extending the shelf‐life of flours and other food products. According to Uyoh, Ita, and Nwofia (2013), moisture content is an index of its water activity important for stability in foods.

**TABLE 1:** Proximate composition of okra seed flour samples

Sample	Moisture (%)	Protein (%)	Fat (%)	Ash (%)	Carbohydrate (%)
*Agbagoma*	9.00 ± 0.01^a^	16.80 ± 0.70^b^	48.00 ± 3.50^a^	7.80 ± 0.10^a^	18.40 ± 0.01^a^
*Balabi*	8.90 ± 1.80^a^	17.40 ± 0.50^a^	47.80 ± 0.20^a^	7.70 ± 0.10^a^	18.20 ± 0.06^a^

Values are means ± standard deviations of triplicate determinations. Values in the same column with different superscript letters are significantly different (*p* < .05).

Protein content was in the range of 16.80%–17.40% for the dried okra seed flour samples (Table 1). The protein content was however higher in *Balabi* (17.40%) than *Agbagoma* (16.80%). These values were generally low when compared to other variety (*iwo agborin*) with protein content of 41.11% (Adelakun et al., 2009). Also, these values were lower than those reported by other researchers including Oyelade, Ade‐Omowaye, and Adeomi (2003) (45.0%) and Calisir and Yildiz (2005) (19.10%). This clearly shows that variety has influence on the protein content. Ali (2010) opined that plant foods that provide about 12% of their calorific value from protein are considered good sources of protein. Although the protein content observed for the two genotypes was low, they met the requirement as a good source of protein.

*Agbagoma* had slightly a higher fat content (48.00%) as compared to *Balabi* which had the lowest (47.80%) (Table 1). However, the fat values were generally higher when compared to other variety (*iwo agborin*) with a fat content of 31.04% (Adelakun et al., 2009). Probably, this indicates that the variety has an influence on the fat content and the higher values observed as compared to literature may be due to the difference in variety and agroecological conditions of plant cultivation.

The ash content ranged from 7.70% to 7.80% with higher value for *Agbagoma* and lower for *Balabi*. In a study on okra pods by Gemede (2016), total ash content was in a range of 5.37–11.30 g/100 g. The total mineral content represents ash content, although minerals represent a small proportion of dry matter, often less than 7% of the total (Olalekan & Bosede, 2010). Thus, it can be assumed that the okra seed flour samples investigated are richer in minerals.

There was no significant difference between the carbohydrate content of the two okra seed flour samples ranging from 18.20% for *Balabi* and 18.40% for *Agbagoma*. However, the carbohydrates values were generally lower than 36.66–50.97 g/100 g reported by Gemede (2016) for eight accessions of okra pod.

### 3.2 Physicochemical properties of okra seed flour

The pH values of the okra seed flour samples ranged from 6.41 to 6.48 with no significant difference between all okra seed flour samples (Table 2). In a study by Ahmed, Shivhare, and Debnath (2002), the authors reported pH 5.08 for green chili puree.

**TABLE 2:** pH and color properties of dried okra seed flour

Sample	pH	Color		
		L*	a*	b*
*Agbagoma*	6.41 ± 0.02^a^	60.29 ± 0.77^b^	0.26 ± 0.06^a^	12.83 ± 0.17^b^
*Balabi*	6.48 ± 0.02^a^	65.37 ± 0.07^a^	0.09 ± 0.02^b^	14.06 ± 0.02^a^

* Values are means ± standard deviations of triplicate determinations. Values in the same column with different superscript letters are significantly different (*p* < .05).

Color value was between the ranges of 60.29 to 65.37 in all okra seed flour samples. The color (L* a* b*) values were significantly higher (*p* < .05) for *Balabi* as compared to *Agbagoma* okra seed flour samples (Table 2). The color brightness coordinate L* measures the degree of whiteness, ranging between black (0) and white (100). The chromaticity coordinate a* measures red when positive and green when negative, and b* measures yellow when positive and blue when negative. Consumer acceptability is affected by the presence of color in starch, which is an indication of low quality (Galvez & Resureccion, 1993). The color brightness (L*) of the *Balabi* seed flour was significantly higher (*p* < .05) than that of the flour from *Agbagoma*. The L* color brightness of both flours can be improved by preventing enzymatic browning by washing the okra seeds with ascorbic acid solution or 1% sodium metabiosulfite solution before sun drying.

The *Balabi* seed flour had the highest bulk density (0.83 g/ml) compared with *Agbagoma* seed flour (0.80 g/ml). In previous work done by Adebowale, Adeyemi, and Osohodi (2005), values obtained ranged from 0.42 to 0.61 g/ml in full‐fat flours and 0.72 to 0.88 g/ml in defatted flours. High bulk density of the dried okra seed flour in this study indicates that they could serve as good thickeners in food products (Abe‐Inge, Arthur, Agbenorhevi, & Kpodo, 2018).

### 3.3 Functional properties of dried okra seed flour

The results of the functional properties of okra seed flour samples are presented in Table 3.

**TABLE 3:** Bulk density and functional properties of dried okra seed flour

Parameters	Dried okra seed flour	
	*Agbagoma*	*Balabi*
Bulk density (g/ml)	0.80 ± 0.01^a^	0.83 ± 0.02^a^
Water absorption capacity (%)	511.64 ± 19.52^a^	504.99 ± 30.10^b^
Oil absorption capacity (%)	88.37 ± 8.62^a^	159.24 ± 20.76^b^
Swelling Power (g/g)	16.37 ± 0.21^a^	14.68 ± 0.77^b^
Solubility Index (%)	14.10 ± 1.27^a^	10.97 ± 1.41^b^

Values are means ± standard deviations of triplicate determinations. Values in the same row with different superscript letters are significantly different (*p* < .05)

Water absorption capacity (WAC) and oil absorption capacity (OAC) of *Agbagoma* were 511.64% and 88.37%, respectively while that of *Balabi* were 504.99 and 159.24%, respectively. There was a significant difference (*p* < .05) between the two varieties. A lower WAC was recorded for irradiated and nonirradiated cowpea flours (110%–113%), full‐fat, and defatted mucuna flour (120%–220%) by Elkhalifa, Schiffler, and Bernhardt (2005) and Adejumo (2013). According to Kinsella (1979), proteins and carbohydrates have great influence on WAC of food due to the presence of hydrophilic components like polar or charged side chains. Interestingly, flours that have the ability to absorb water well and swell for improved consistency in food (high WAC) have beneficial applications in dough, processed meats, and custards.

The oil absorption capacity (OAC) of food determines the mouth‐feel, flavor retention as well as shelf‐stability of baked or fried foods and especially meat products. According to Adebowale and Lawal, (2004), OAC of food matrices and absorption of oil is influenced by proteins when the protein surfaces increase the hydrophobic interaction of proteins with flavor compounds as well as the binding of food to the inner walls of the mouth during chewing.

The swelling power of the okra seed flour ranged from 14.68 to 16.37 g/g. The swelling capacity values were not in accordance with (Adetuyi et al., 2009) who examined maize–soybean flour blends. The low swelling power value could be attributed to the low carbohydrate content of the okra seed flour because the ability to swell is a function of the carbohydrate content.

There was significant difference (*p* < .05) between okra seed flour samples in terms of solubility index. Often, the solubility and swelling power are influenced by the water‐binding capacity of the flour sample, which is a function of proteins and carbohydrates present in the flour (Abe‐Inge et al., 2018; Baah, Oduro, & Ellis, 2005; Dossou, Agbenorhevi, Alemawor, & Oduro, 2014).

### 3.4 Pasting properties of okra seed flour

The pasting property parameters of *Agbagoma* seed flour were higher than that of the *Balabi* seed flour (Table 4). Adams, Wireko‐Manu, Agbenorhevi, and Oduro (2019) reported peak viscosity as an indication of extent of granule swelling and the strength of the associative forces between the molecules of the flour.

**TABLE 4:** Pasting properties of okra seed flour samples

Parameters	Dried okra seed flour	
	*Agbagoma*	*Balabi*
Peak viscosity (cP)	759.00 ± 28.28^a^	85.50 ± 14.85^b^
Trough (cP)	337.00 ± 1.41^a^	74.00 ± 8.49^b^
Breakdown (cP)	422.00 ± 26.87^a^	11.50 ± 6.36^b^
Final viscosity (cP)	437.50 ± 18.19^a^	106.50 ± 3.54^b^
Setback (cP)	100.50 ± 16.77^a^	32.50 ± 4.95^b^
Peak time (min)	1.97 ± 0.42^b^	6.07 ± 0.19^a^
Pasting temperature (°C)	50.20 ± 0.00^a^	0.23 ± 0.04^a^

Values are means ± standard deviations of triplicate determinations. Values in the same row with different superscript letters are significantly different (*p* < .05).

The high peak viscosity of the *Agbagoma* seed flour shows that water molecules penetrate easily causing enormous granular swelling, which in turn weakens the associative forces of the flour hence makes them susceptible to breakdown as compared to the *Balabi* seed flour with lower peak viscosity (Etudaiye, Nwabueze, & Sanni, 2009).

Although the trough viscosity is the ability of starch to withstand long duration of hot temperature during processing or heating, the *Agbagoma* seed flour with high hot paste stability would preferably be used in food processing than *Balabi* seed flour.

According to Olufunmilola, Jacob, and Tajudeen, (2009), flours with low break down are more stable under hot conditions and have strong cross‐linking within the granules. It therefore implies that there is stronger cross‐linking within the granules of the *Balabi* seed flour. *Agbagoma* seed flour recorded higher final viscosity, which suggests its ability to form gel/paste during cooling (Shimelis, Meaza, & Rakshit, 2006) and more suitable in the processing of food products such as sauces, soups, and dressings.

As the setback value is a measure of gel stability and potential of retrogradation, the low setback value of the *Balabi* seed flour suggests its high resistance to retrogradation than the *Agbagoma* seed flour with a higher setback value. *Balabi* seed flour can be incorporated into wheat flour in the production of pastries such as bread, pie, and others (Adams et al., 2019).

## 4 CONCLUSION

The dried okra seed flours (*Agbagoma* and *Balabi*) revealed a high content of fat and proteins. The high protein content could be a valuable protein supplement for cereals based foods. The pH values of the okra seed flour were within the recommended range which is ideal for individuals with ulcers‐related problems. The functional properties of okra seed flour were appreciable that could be exploited in food formulations such as cereals and wheat flour. All pasting properties except peak time and pasting temperature of *Agbagoma* seed flour were higher than that of *Balabi* seed flour suggestion that *Balabi* seed flour could be used as composite for baby foods because of its low viscosity whereas flour from *Agbagoma* could be used as composite for high viscous food.

## CONFLICT OF INTEREST

All the authors hereby state that there is no conflict of interest regarding this publication.
